# Cross-National Analysis of Consumer Preferences for Organic Food in Portugal, Spain, and Greece: Socio-Demographic Drivers and Attribute Importance

**DOI:** 10.3390/foods15010155

**Published:** 2026-01-03

**Authors:** Teresa Madureira, Fernando Nunes, Fernando Mata, Mariastela Vrontaki, Athanasios Manouras, Michalis Koureas, Eleni Malissiova, José Veiga

**Affiliations:** 1 Centre for Research and Development in Agrifood Systems and Sustainability (CISAS), Instituto Politécnico de Viana do Castelo (IPVC), 4900-347 Viana do Castelo, Portugal; teresa@esa.ipvc.pt (T.M.); fnunes@esa.ipvc.pt (F.N.);; 2 Estação Zootécnica Nacional, Instituto Nacional de Investigação Agrária e Veterinária, 2500-424 Vale de Santarém, Portugal; 3 Food of Animal Origin Laboratory, Animal Science Department, University of Thessaly, 41500 Larisa, Greece; svrontaki@uth.gr; 4 Food Chemistry, Biochemistry and Technology Laboratory, Nutrition and Dietetics Department, University of Thessaly, 42132 Trikala, Greece; amanouras@uth.gr; 5 Department of Hygiene and Epidemiology, Faculty of Medicine, University of Thessaly, 41500 Larisa, Greece; mkoureas@uth.gr; 6 Escola Superior de Tecnologia e Gestão (ESTG), Instituto Politécnico de Viana do Castelo (IPVC), 4900-347 Viana do Castelo, Portugal; jmcveiga@estg.ipvc.pt

**Keywords:** organic food, consumer preferences, Greece, Portugal, Spain, multivariate analysis, sustainability

## Abstract

Consumer demand for organic products has grown substantially in Southern Europe, driven by health, environmental, and ethical concerns. Understanding cross-country differences in attribute preferences and sociodemographic influences is critical to inform marketing strategies and policy interventions targeting organic food consumption. To perform a comparative study across Portugal, Spain, and Greece, regular organic consumers were surveyed (250 per country) using a culturally adapted Best–Worst Scaling questionnaire. Socio-demographic variables and ten organic food attributes were analysed using MANOVA, Kruskal–Wallis tests, PCA, and cluster analysis. Spanish and Portuguese consumers prioritised health, environmental impact, absence of GMOs, and certification, while Greeks emphasised price, appearance, taste expectation, and nutrition. Age, gender, and education influenced attribute importance differently across countries, revealing distinct national consumption patterns and preferences. Findings highlight substantial heterogeneity: health and environmental attributes dominate in Portugal and Spain, reflecting strong certification and sustainability awareness, whereas Greek consumers focus on value, sensory qualities, and nutrition, indicating lower organic uptake and stronger price sensitivity. Older and more educated consumers valued certification and provenance, women emphasised health and environmental benefits, and men responded more to convenience and status cues. These patterns suggest that marketing and policy strategies should combine universal motivators with tailored approaches addressing national, demographic, and cultural differences to enhance organic consumption. Cross-country differences reveal the need for context-specific interventions promoting organic food while leveraging common health and sustainability drivers.

## 1. Introduction

Driven by a complex interplay of socio-economic, environmental, and health-related concerns, global consumer demand for organic products has witnessed a marked and sustained increase in recent years. This shift reflects broader societal trends toward sustainability and ethical consumption, necessitating the development of more resilient and environmentally responsible agri-food supply chains [1,2]. Within the European Union (EU), this trend is particularly pronounced, as evidenced by a substantial expansion in certified organic agricultural land pushed by the Common Agricultural Policy. Research has demonstrated that EU citizens support these policies [3,4]. According to Eurostat, the total organic area in the EU grew by 79% between 2012 and 2022, reaching approximately 16.9 million hectares in 2022 [5].

Southern European countries, specifically Greece, Portugal, and Spain, have played a significant role in this expansion. In Greece, the organic agricultural area nearly doubled over the decade, increasing from 462,618 ha in 2012 to 924,853 ha in 2022. Portugal demonstrated an even more pronounced growth trajectory, with organic farmland expanding from 200,833 ha to 759,977 ha over the same period. Similarly, Spain’s organic area more than doubled, rising from 1,167,362 ha in 2012 to 2,349,475 ha in 2022. These developments reflect not only domestic policy support and EU-level incentives but also a growing alignment with consumer preferences for sustainable food systems [6]. Notably, in 2022, the combined organic area of Greece, Portugal, and Spain accounted for 25.8% of the EU’s total organic farmland, underscoring their pivotal role in advancing the EU’s ecological transition in agriculture [5].

The intensification of agricultural production over recent decades has been accompanied by the widespread and, at times, indiscriminate use of herbicides, pesticides, synthetic fertilisers, genetically modified organisms (GMOs), antibiotics, and other agrochemical inputs. This trajectory has prompted significant concern within the scientific community regarding the detrimental impacts of such practices on environmental sustainability, biodiversity, soil health, and ecosystem resilience [7,8]. In response to mounting evidence and increasing global awareness, consumers are becoming more cognizant of the broader implications of their dietary choices. These evolving attitudes are reshaping consumption patterns, with a growing segment of the population actively seeking products that align with environmental sustainability principles [9,10].

Within the EU, the production, processing, and labelling of organic products are regulated by Regulation (EU) 2018/848, which establishes comprehensive standards aimed at promoting environmentally sound and socially responsible agricultural practices [11].

Many consumers perceive organic foods as healthier, safer, and more nutritious than their conventional counterparts, which significantly influences purchasing decisions [12,13,14,15]. In recent years, heightened demand for organic products has also been fuelled by recurring food safety scandals widely covered in the media, which have amplified public concern about the integrity and safety of conventional food systems [16,17]. Moreover, the COVID-19 pandemic further intensified health consciousness among consumers, leading to a discernible shift in purchasing patterns toward products perceived as more natural and less chemically intensive [18].

Beyond health and safety, several other factors shape consumer attitudes toward organic food. Sensory attributes such as taste, texture, and appearance play an important role in product selection [19,20], as does the presence of organic certification labels. The efficacy of these labels in influencing consumer behaviour largely depends on the degree of recognition and trust they command [21]. Socio-demographic and structural variables (including urbanisation, education level, age, gender, and income) also contribute to the heterogeneity of consumer preferences [22].

A range of economic and structural barriers also significantly shape consumer behaviour toward organic food in Greece, Portugal, and Spain. These include widespread mistrust in organic certification schemes [12,15,23], the relatively high price of organic products compared to the conventional alternative [24,25,26], and the limited availability of organic food items in mainstream retail outlets, particularly supermarkets [13,27,28].

By systematically examining the multifaceted cross-national differences in consumer perceptions and priorities regarding organic food in Greece, Portugal, and Spain, this study endeavours to generate comprehensive and contextually grounded insights. The knowledge, attitude [29], and practice framework is used to systematically assess what people know, how they feel, and how they behave in relation to organic food. By identifying gaps and misalignments among these three components, practitioners can design more targeted and effective interventions. This research aims to inform the design and implementation of targeted marketing strategies that resonate with diverse consumer segments, while also guiding public policy frameworks toward greater inclusivity and effectiveness.

This article seeks to consolidate and expand upon previous analyses conducted in Portugal [15], Spain [13], and Greece [14], aiming to explore the similarities and divergences in organic food consumer profiles in the three countries.

## 2. Materials and Methods

The questionnaires employed in these studies were meticulously translated into the respective national languages to preserve semantic accuracy and cultural appropriateness. To ensure clarity, relevance, and comprehensive understanding across diverse participant groups, a rigorous pre-testing phase was conducted in each country. This process involved pilot testing with representative samples, allowing for refinement of wording, adaptation of culturally specific references, and validation of the questionnaire’s overall comprehensibility.

Each questionnaire included a detailed informed consent section, which participants were required to review and explicitly acknowledge before proceeding with the survey. This ensured that respondents were fully aware of the study’s purpose, their rights, and the voluntary nature of their participation. Ethical approval for the study in all countries was obtained from the Department of Animal Science at the University of Thessaly, Greece (reference number 30515/24/TEZP), underscoring adherence to rigorous ethical standards in cross-national research.

### 2.1. Generic Description of Sample Preparation and Data Collection in Portugal, Spain, and Greece

In the three national studies, conducted in Portugal, Spain, and Greece, a quota sampling strategy was employed to ensure demographic representativeness. Quotas were established proportionally according to key variables: region of residence, age group, gender, and educational attainment.

Participants were initially invited to complete a structured online questionnaire. However, to fulfil all predefined quotas and guarantee adequate representation across demographic strata, face-to-face interviews were conducted when necessary. To maintain the focus on regular organic food consumers, individuals who reported consuming fewer than three organic products per week were excluded from the sample. A total of 750 questionnaires were administered (250 in each country) with data collection taking place between 2020 and 2023.

The questionnaire was structured in two main sections. The first section collected socio-demographic and behavioural information through six classification variables: gender, age, place of residence, education level, presence of children under 18 years old in the household, and usual place of purchase of organic products. This information is summarised in Table 1.

The second section assessed the perceived importance of ten key attributes associated with organic food products: price, more natural appearance, certification warranty, origin, expected better taste, availability, health benefits, environmental impact, nutritional value, and absence of GMOs. This evaluation was conducted using the Best–Worst Scaling (BWS) method, which allows for the identification of relative preferences by asking respondents to indicate the most and least important attributes across a series of choice sets.

According to this framework, three credence attributes were considered: health benefits (‘Health’) [30,31,32,33], environmental impact (‘Environment’) [34,35,36,37,38,39], and nutritional value (‘Nutrition’). [10,40,41,42,43,44]; one experience attribute, namely expectation of better taste (‘Expectation’) [45,46,47]; and six search attributes, which include ‘Price’ [32,40,48,49,50,51,52], more natural appearance (‘Aspect’) [1,53,54,55,56], certification warranty (‘Certification’)) [21,57,58,59,60,61,62], origin (‘Origin’) [63,64,65,66,67], availability (‘Buying easy’) [9,32,40,49,68,69], and absence of GMOs (‘NoGMO’) [1,70,71].

### 2.2. Best–Worst Scaling Methodology

The Best–Worst Scaling (BWS) method, also known as Maximum Difference Scaling (MaxDiff), represents a significant advance in preference elicitation techniques.

In the case of organic products specifically, BWS is particularly effective because consumer decisions often involve balancing multiple, sometimes competing, criteria. Choices between organic and conventional alternatives frequently hinge on factors such as price premiums, perceived health benefits, environmental impacts, and certification labels. BWS facilitates direct prioritisation of these factors, enabling researchers to move beyond generic ordinal measures (e.g., “highly sustainable”) to quantify trade-offs with precision. Our prior research ([13,14,15]) has confirmed the methodological advantages of BWS for analysing regional preference heterogeneity. The current comparative study across Portugal, Spain, and Greece leverages these strengths to explore cultural determinants of consumer priorities and to uncover actionable insights for advancing the organic food market in diverse socio-economic contexts.

### 2.3. Statistical Analysis

To verify the distribution of the different classification variables within the three countries, cross-tabulations were performed, using Pearson’s test of independence to assess significant differences.

The different organic food attributes were then tested for significant differences between countries (Greece, Portugal, Spain) with a MANOVA via Pillai’s trace test, as this was shown to be more robust in tolerating deviations from the homogeneity of variances and normal distribution of residuals. The prerequisites for the parametric tests were tested via Kolmogorov–Smirnov tests for the normal distribution of the residuals and via Levine’s test for the homogeneity of variances. As these prerequisites were constantly violated, univariate non-parametric Kruskal–Wallis tests were performed. Pairwise comparisons of medians were achieved after a Bonferroni correction for multiple tests.

To complement the univariate tests, data were also explored through multivariate analysis, namely, using principal components analysis with the production of a biplot. A cluster analysis was also performed based on the cases, with the Ward aggregation method together with the Manhattan method to calculate the linkage distances.

Finally, to contrast the key attributes with the classification variables, linear-scale response, generalised linear models were implemented using the former as dependent and the latter as independent variables. The two-way interactions between the countries and the demographic variables were also entered initially in the models. A backward stepwise procedure was implemented for the selection of variables. The models’ omnibus test used was the likelihood ratio chi-squared, and the tests of model effects, together with the hypothesis tests for the parameter estimation, were achieved via the Wald chi-squared test. The PCA and cluster analyses were performed using R for Mac OS version 4.4.2 X GUI 1.81 Big Sur Intel build (8462). All the other analyses were using IBM Corp.^®^ SPSS^®^ Statistics, Armonk, NY, USA. Version: 29.0.2.0 (20).

## 3. Results

### 3.1. Relationship Between Countries and Classification Variables

The relationship between the three countries, Portugal, Spain, and Greece, and the classification variables is summarised in Table 2. The tables of contingency can be consulted in Appendix A.

As can be observed, the only variable independent of ‘Country’ is the presence of children under 18 in the household. All other variables show a certain degree of association with the country. Youth and older age groups are more highly represented in Greece, whereas intermediate age groups are more prevalent in both Portugal and Spain. With respect to ‘Gender’, Greece shows a higher representation of women. Regarding ‘Education level’, lower levels of education are more common in Greece, while higher levels of education are more prevalent in Portugal. These are sociodemographic variables that are not necessarily collected in accordance with the actual population distributions within each country. Therefore, further country-specific interpretation of the results requires careful consideration, as the effect of the country may be masked by one or more of these sociodemographic variables. Preferences for ‘Places to buy’ organic products also differ between countries, with respondents in Portugal favouring markets, those in Spain preferring traditional shops, and those in Greece more frequently supermarkets.

### 3.2. Relationship Between Countries and the Key Attributes

**Figure 1:**
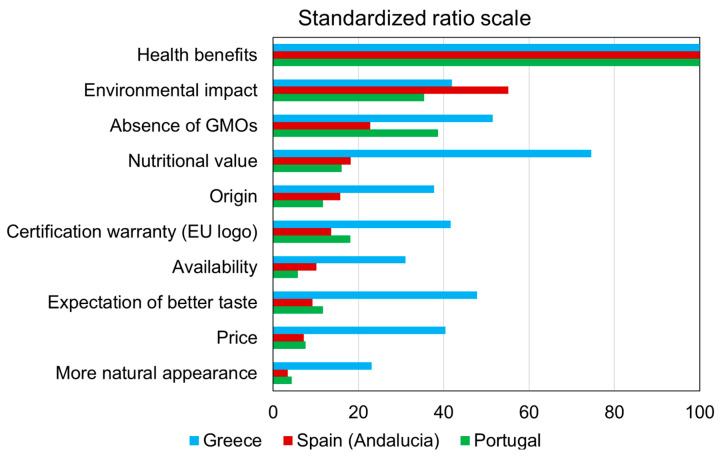
The 10 attributes of organic products displayed on a standardised ratio scale for the three countries.

To make a comparison between the three countries and analyse the most and least preferred attributes, we use the ten attributes based on the standardised ratio scale index (Figure 1).

Consumers across the three countries consistently identified health benefits as the most salient driver of organic product consumption. With the highest-ranked attributes standardised to a reference value of 100 (as the standardised ratio scale functions as a relative indicator), the graphical representation of the remaining attributes provides a clear illustration of cross-country heterogeneity. The attributes displaying the greatest variability, in descending order, include a ‘More natural appearance’, ‘Price’, ‘Expectations of better taste’, ‘Nutritional value’, and Availability’. In contrast, minimal heterogeneity was observed for the ‘Absence of GMOs’ and ‘Environmental impact’, which appear to be more uniformly valued across countries.

Overall, the visual patterns suggest moderate heterogeneity in organic consumer preferences among Portugal, Spain, and Greece, with Greek consumers demonstrating distinct prioritisation profiles relative to their Portuguese and Spanish counterparts.

A Spearman correlation matrix was applied to the ten attributes, confirming that in 80% of cases, *p* < 0.05, indicating significant correlations between attributes.

The MANOVA was found to be significant (Pillai’s trace = 1.621, F(27, 2223) = 96.80, *p* < 0.001), clearly demonstrating differentiation between the three countries with respect to the importance assigned to the various attributes of organic food. Table 3 presents the results of the Kruskal–Wallis tests applied to each attribute to distinguish the countries’ relative positioning.

As shown in the results presented in Table 3, Greek consumers attach particular importance to *Price, Aspect, Expectation*, and *Nutrition*. Portuguese and Spanish consumers are more closely aligned with each other in their emphasis on *Health, Environment*, and *NoGMO*. The Spanish differ from the Portuguese mainly by assigning greater importance to *Origin*, whereas Portuguese consumers place notably high importance on *Certification*.

Conversely, Greek consumers attach lower levels of importance to *Environment* and *NoGMO*. Portuguese and Spanish consumers, in contrast to Greeks, assign lower importance to *Price* and *Aspect*. Compared with both Greeks and Spaniards, Portuguese consumers place less importance on *Buying Easy*, while Spanish consumers show lower levels of importance for Expectation relative to the other two countries.

**Figure 2:**
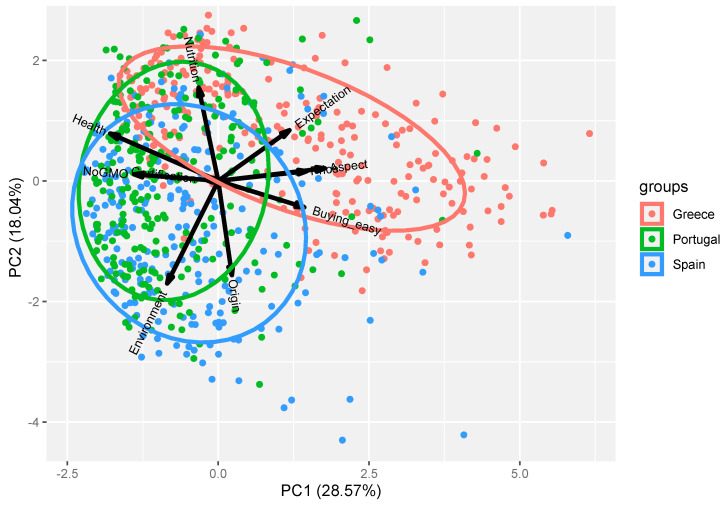
Biplot projecting the consumers of the three countries in the bi-dimensional space created by the 2 two main principal components (PC) extracted from the dataset. The directional vectors related to each of the attributes are also represented. Note the overlap between the vectors ‘Aspect’ and ‘Price’, and also between ‘NoGMO’ and Certification’.

The Principal Components Analysis (PCA) enabled the extraction of two principal components, which together explain only 46.61% of the variance in the dataset (28.57% + 18.04%). Nevertheless, the projection of respondents onto this two-dimensional space reveals a clear clustering of the three countries (Figure 2).

**Figure 3:**
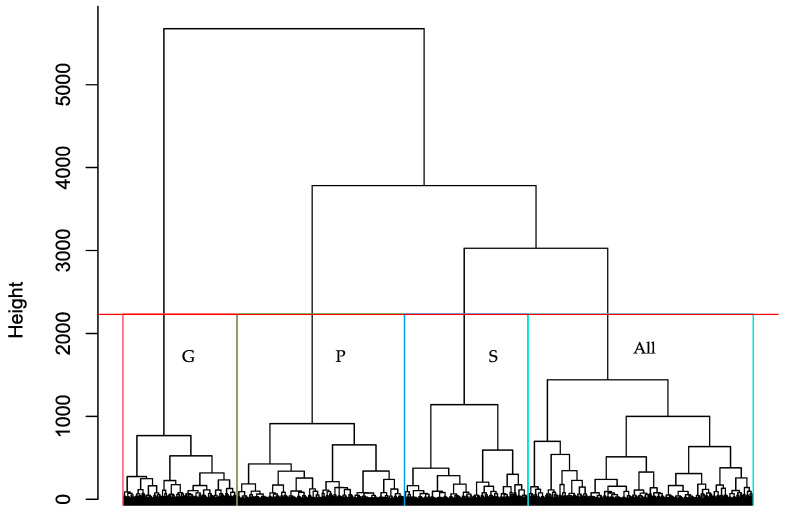
Cluster dendrogram with the agglomeration of the consumers. G: Greece; P: Portugal; S: Spain; All: The three countries. The cluster cut-off line indicates the 3 countries and a mixture of all.

This result reinforces the patterns observed in the Kruskal–Wallis analysis, showing a clear grouping of the three countries according to the levels of importance attributed to the key organic food attributes. Furthermore, this grouping is corroborated by the cluster analysis presented in Figure 3, where three distinct clusters emerge, each predominantly composed of consumers from a single country. A fourth cluster comprises consumers from all three countries and does not exhibit a dominant representation from any of them.

### 3.3. Impact of the Classification Variables on the Key Attributes

The impact of the variables on the key attributes can be viewed in Table 4, Table 5, Table 6 and Table 7.

As can be observed, *Price* is mainly influenced by *Age group* and *Education*. Older individuals are more concerned about price than younger individuals, and those with lower levels of education also demonstrate greater sensitivity to the price of organic food. The interaction *Country* × *Education* reveals that young Greeks are the most concerned about the price of organic food, whereas older, yet still economically active, Spaniards (aged 55 to 69) are the least concerned.

With regard to *Aspect*, this attribute is primarily influenced by *Gender* and the *Country* × *Education* interaction. Men place greater value on the more natural appearance of organic food than women. This pattern is also observed among less educated Greek consumers, particularly those with only basic and/or primary schooling.

*Certification* is mainly influenced by *Age group*, the presence of children under 18 in the household, and the *Country* × *Age group* interaction. Older individuals assign the highest levels of importance to organic certification. Respondents without *Children under 18 in the household* also give greater importance to *Certification*. Greek consumers, particularly those in the older age group, assign the lowest levels of importance to *Certification*. Spaniards aged 35 to 54 display a similar pattern.

Regarding the country of *Origin* of organic food, the only significant parameter is the *Country* × *Education* interaction. No consistent trend emerges. Spaniards with only primary education show the highest levels of concern for the product’s *Origin*. They are followed by Greeks with primary education, as well as those with a Bachelor’s degree. Portuguese and Spaniards with a Bachelor’s degree also show heightened concern. Lower concern is observed among Portuguese consumers with basic or secondary education.

*Expectation* is mainly influenced by *Age group*, the presence of *Children under 18 in the household*, the preferred purchasing location, and the *Country* × *Gender* interaction. Younger individuals (up to 54 years old) assign higher levels of importance to the expectation that organic foods will taste better. Individuals without *Children under 18 in the household* also place greater importance on this attribute. Higher levels of importance are likewise observed when organic food is purchased through home sales or at markets.

Greek consumers (particularly men) show the highest levels of expectation regarding the taste of organic foods, whereas Spanish women exhibit the lowest levels of expectation.

*Buying Easy* is mainly influenced by *Gender* and by the *Country* × *Gender* interaction. Men place greater value on the availability and ease of purchasing organic food, particularly Spanish and Greek men.

*Health* is primarily influenced by *Gender* and the *Country* × *Education* interaction. Women attribute greater importance to the health benefits of organic foods than men. Greek consumers with lower levels of education assign the least importance to health benefits, a pattern also observed, however less markedly, among Spaniards educated up to Bachelor’s level.

*Environment* is mainly influenced by the *Country* × *Gender* and *Country* × *Education* interactions. Greek men, as well as Spanish men and women, show lower levels of concern regarding the environmental impact of organic food production than other combinations of country and gender. Greeks with only primary or secondary (high school) education also express lower levels of concern, whereas Spaniards with primary or secondary schooling display greater concern.

*Nutrition* is primarily influenced by the *Country* × *Gender* interaction. Greek consumers (particularly women) assign the highest levels of importance to the nutritional aspects of organic foods. Spanish and Portuguese consumers, especially women, place comparatively lower importance on this attribute.

The importance attributed to the absence of GMOs in organic foods is mainly influenced by *Gender*, *Buying place*, and the *Country* × *Education* interaction. Women value this attribute more than men. Lower importance is assigned to the *Absence of GMOs* when organic food is purchased at markets. Portuguese consumers with either basic education or a Bachelor’s degree show higher levels of concern about the *Absence of GMOs*, whereas Greeks generally assign lower levels of importance. Spaniards educated up to Bachelor’s level also place relatively low importance on the *Absence of GMOs* in organic food.

## 4. Discussion

The findings of this study provide new insights into the heterogeneity of consumer preferences for organic products across three Southern European countries: Portugal, Spain, and Greece. The present comparative analysis highlights both commonalities and divergences in the sociodemographic determinants and key attributes driving organic food consumption in the region.

Age exerts a consistent, yet context-sensitive, influence on organic-product preferences across Portugal, Spain and Greece. In our pooled sample, older respondents showed greater price sensitivity and placed relatively more weight on certification and provenance, whereas younger cohorts prioritised sensory expectations (taste, appearance) and, to a greater extent in some contexts, environmental and identity-related motives. These patterns align with the mixed evidence in the literature: age often moderates the trust–value relationship and alters how consumers process altruistic versus egoistic claims about food [15,72,73]

However, equality and divergence between the three countries are equally instructive. Portugal and Spain displayed similar age-linked profiles in which health and environmental credence attributes remained highly salient across most cohorts, consistent with national studies that report comparatively strong certification and environmental concerns among middle-aged and older consumers. Greece differed: price, appearance and taste expectations were especially prominent among both younger and older segments in our Greek sample. Greeks show lower overall organic uptake but stronger sensitivity to value and sensory cues [14].

Mechanisms that plausibly explain these age × country interactions include life-stage (household composition and retirement), disposable income and education (which mediate price elasticity), and cohort-specific values (e.g., stronger climate concern among younger generations) [74,75]. The COVID-19 pandemic amplified health salience across ages but did not eliminate national differences: pandemic-driven demand increased organic purchases broadly, yet price pressures and retail structure (supermarkets versus direct sales) modulated uptake differently in each country [76,77].

For policy and marketing, this implies a dual strategy: maintain pan-Mediterranean messaging that emphasises universal motivators (health, safety) while tailoring campaigns by life-stage and national context. For older and lower-income segments, particularly in Greece, messages should stress value and certification credibility; for younger cohorts, especially in Portugal and Spain, emphasise sustainability credentials, taste, and provenance as identity markers. Such segmentation is supported by broader reviews of organic-food drivers and by studies linking demographic moderators to purchase intentions [76].

Gender markedly influences motivations, attribute priorities, and purchasing behaviour for organic food. Across many contexts, women are consistently more likely to purchase organic products and to rank health- and environment-related motives higher than men [40,78]. Empirical work shows women report stronger environmental concern and health consciousness, which are mechanisms commonly invoked to explain a female “premium” for organic purchases (socialisation/role theories and ecofeminist perspectives) [79,80,81].

At the same time, several studies report that men, when they do buy organic, can display a higher willingness-to-pay for premium or convenience formats; men’s purchase decisions often respond more to status, price signalling, or situational factors than to health narratives [79]. Meta-analytic and large-sample research, therefore, depicts a nuanced picture: higher female prevalence of regular organic buying but gendered differences in the strength and nature of motives and price sensitivity [82].

Our three-country comparison aligns with these patterns but also reveals important national differences. In Portugal and Spain, female respondents place relatively greater weight on health, environment and absence of GMOs. In Greece, however, gender effects interact with education and age: Greek men in our sample gave larger emphasis to product appearance and availability, while Greek women rated nutrition highly—a pattern consonant with earlier Greek consumer literature that stresses product-type and traditional diet effects [12,83]. 

Policy and marketing also have implications for gender analysis choices for organic foods. Communications stressing health and environmental benefits resonate more strongly with female segments in all three countries; labelling and credible certification, therefore, remain crucial [74]. Also, because men may be more price-sensitive or oriented to convenience and status cues in some contexts, strategies that emphasise value (promotions, bundle offers) and convenience (availability in mainstream retail, ready-to-eat formats) can broaden male uptake. Finally, in terms of national tailoring matters, Greece’s stronger role of appearance/nutrition suggests that sensory and culinary messaging (taste, traditional recipes using organic ingredients) could be relatively more effective there, whereas Portugal and Spain may respond better to campaigns emphasising certification, provenance and environmental credentials [13].

Educational attainment significantly influences consumer behaviour towards organic food, with higher levels of education often correlating with increased awareness and preference for organic products. Studies indicate that individuals with higher education levels are more likely to purchase organic foods due to greater health consciousness, environmental awareness, and knowledge of food safety issues [84].

In Portugal, education plays a pivotal role in shaping organic food consumption patterns. Consumers with higher education levels exhibit a greater understanding and preference for organic products [84]. This trend is attributed to increased awareness of health and environmental benefits associated with organic foods. Additionally, educated consumers in Portugal are more likely to seek information about food origins and production methods, leading to more informed purchasing decisions [85].

Similarly, in Spain, education influences organic food consumption. Consumers in Spain with higher education levels are more willing to pay a premium for organic products, driven by concerns over health and environmental sustainability [86,87]. Furthermore, a study [87] in Tenerife revealed that educated consumers are more likely to prioritise organic food in their diets, reflecting a broader European trend

In Greece, education also impacts organic food consumption, albeit with some regional variations. Greek consumers with higher education levels place significant importance on the nutritional benefits and environmental impact of organic foods. However, factors such as product appearance and availability were also influential, indicating that education interacts with other socio-cultural factors in shaping consumption patterns.

Comparative analyses across these countries reveal that while the general trend shows that higher education levels correlate with increased organic food consumption, differences exist. In Portugal and Spain, education primarily enhances awareness and willingness to pay for organic products. In contrast, in Greece, education interacts with regional preferences and cultural factors, influencing the importance placed on various attributes of organic foods.

The presence of children under 18 in the household also significantly influences organic food preferences. In Portugal, households with children demonstrate a higher propensity to purchase organic products, driven by health considerations and concerns over pesticide exposure [13]. Similarly, Spanish families with children are more likely to prioritise organic food, reflecting a growing awareness of the nutritional benefits and safety of organic products for young consumers [15]. In contrast, Greek households with children exhibit less pronounced preferences for organic food, potentially due to economic constraints and limited availability of organic options [14]. While families with children generally show a preference for organic products, the strength of this preference varies across countries, influenced by factors such as economic conditions, cultural attitudes towards food, and the availability of organic products in the market.

Consumer preferences for organic food purchasing locations exhibit differences across the studied countries. In Portugal, a significant proportion of consumers prefer purchasing organic products from supermarkets and hypermarkets, driven by convenience, product variety, and perceived safety. Similarly, in Spain, large retail chains are popular among organic food shoppers. The Greek consumers often favour local markets and direct sales from producers, influenced by economic constraints and a stronger tradition of local food sourcing.

### 4.1. Study Outcomes

A key contribution of this study is the insight gained from the simultaneous comparison of Portugal, Spain and Greece, which goes beyond what can be inferred from separate national analyses. While age, gender, education and household composition emerge as important predictors in all three countries, the comparative approach shows that their influence differs systematically across contexts. For instance, age reinforces trust in certification and environmental claims in Portugal and Spain, but in Greece, it heightens sensitivity to price and sensory attributes. Similarly, gender effects that might appear broadly uniform in single-country studies are shown to interact differently with education, culture and retail structures, particularly in Greece, where appearance, availability and traditional food norms play a stronger role than in the Iberian countries.

The comparison also reveals that education and family status operate through distinct mechanisms rather than exerting uniform effects. Higher education increases willingness to pay and environmental awareness in Portugal and Spain, but in Greece, it reshapes trade-offs between nutrition, appearance and availability. Likewise, the presence of children strongly activates health-related organic purchasing in Portugal and Spain, but much less so in Greece, where economic constraints and market access limit uptake. Overall, the three-country comparison highlights context-dependent patterns that would remain hidden in national studies, strengthening both the explanatory value of the findings and their relevance for policy and marketing.

The study is important because it shows that preferences for organic food in Southern Europe are shaped not only by sociodemographic factors, but by how these interact with national contexts. Although age, gender, education and household composition influence behaviour in all three countries, the comparative analysis demonstrates that their effects differ across Portugal, Spain and Greece, helping to explain persistent differences in organic uptake that would not be evident from separate national studies.

These results can be used to inform more effective policy and market strategies. They support combining common health and safety messages with country-specific interventions, such as addressing price and availability barriers in Greece or emphasising sustainability and certification in Portugal and Spain, thereby improving the targeting and effectiveness of organic food promotion.

### 4.2. Study Limitations

Despite the insights provided, this study presents some limitations. The use of quota sampling, not stratified within the countries and geographically limited, combined with self-reported data, may introduce selection and reporting biases, potentially limiting the generalisability of the findings beyond regular organic consumers. Also, while the Best–Worst Scaling methodology allows robust preference elicitation, it captures stated rather than revealed behaviours, which may not fully reflect actual purchasing patterns in real-world retail environments. Another aspect is that the study focused on ten pre-selected attributes; other potentially influential factors, such as ethical production, packaging, or local origin, were not included and may affect consumer decision-making. Additionally, the cross-sectional design precludes causal inference and may not capture dynamic changes in preferences over time, particularly in response to evolving economic conditions or policy interventions. As cited during the Discussion, the pandemic years observed a duplication of organic food consumption in Europe. Finally, although the three countries provide valuable comparative insights for Southern Europe, results may not be directly transferable to other European regions with different cultural, socio-economic, or retail structures.

### 4.3. Future Research Directions

Future research should consider longitudinal approaches to track changes in consumer preferences, integrate observed purchasing data to validate stated choices, and expand the range of attributes to include ethical, social, and experiential dimensions. Comparative studies including additional Southern and Northern European countries could further elucidate cultural and structural determinants of organic food consumption, while segmentation analyses focusing on minority and underrepresented groups would enhance understanding of equity and inclusivity in sustainable consumption patterns.

## 5. Conclusions

This comparative analysis highlights the differences and context-dependent nature of organic food consumption patterns across Portugal, Spain, and Greece. While health and environmental concerns consistently emerge as significant motivators for organic food purchases in Portugal and Spain, Greek consumers exhibit a more pronounced sensitivity to price, product appearance, and sensory expectations. These differences are influenced by a complex interaction of sociodemographic factors, as well as cultural and economic contexts unique to each country.

The study highlights the importance of tailoring marketing strategies to align with the specific preferences and values of consumers in each country. In Portugal and Spain, emphasising the health and environmental benefits of organic products, along with credible certification and origin information, may appeal more effectively to consumers. In contrast, in Greece, marketing messages that focus on value, convenience, and sensory attributes could better capture consumer interest.

## Figures and Tables

**Table 1:** Structure of the sample.

Classification Variable (Factor)	Level
Age group	18–34
	35–54
	55–69
	70+
Gender	Man
	Woman
Education level	Primary
	Basic
	High School
	BSc
	MSc/PhD
Children < 18 in the household	Yes
	No
Best place to buy Organic	Markets
	Dedicated stores
	Supermarkets
	Home sale
	Traditional stores

**Table 2:** Crosstabulation Person’s chi-square test of independence applied to countries and each of the classification variables. The tables of contingency resulting from this analysis can be consulted in Appendix A as indicated.

Model	Person’s χ^2^	df	*p*-Value	Table of Contingency
Country × Age group	107.56	6	<0.001	Table A1
Country × Gender	10.62	2	=0.005	Table A2
Country × Education level	840.67	8	<0.001	Table A3
Country × Children < 18	1.383	5	=0.501	Table A4
Country × Buying place	119.78	8	<0.001	Table A5

**Table 3:** Results of the Kruskal–Wallis tests on the importance given to each of the different key attributes by the three countries: Medians with different letters in superscript are indicative of significant differences after a Bonferroni correction for multiple tests (*p* < 0.05). H is the test value.

Key Attributes	H	df	*p*-Value	Greece	Portugal	Spain
Price	102.05	2	<0.001	7.20 ^b^	1.51 ^a^	1.36 ^a^
Aspect	164.58	2	<0.001	3.29 ^b^	0.47 ^a^	0.24 ^a^
Certification	14.11	2	<0.001	7.91 ^a^	10.19 ^b^	5.49 ^a^
Origin	10.59	2	=0.005	7.30 ^b^	4.76 ^a^	7.88 ^b^
Expectation	110.82	2	<0.001	9.00 ^b^	3.17 ^a^	2.24 ^a^
Buying easy	78.62	2	<0.001	4.81 ^b^	0.71 ^a^	2.45 ^b^
Health	21.73	2	<0.001	21.50 ^a^	25.81 ^b^	25.14 ^b^
Environment	214.30	2	<0.001	7.42 ^a^	13.31 ^b^	19.02 ^c^
Nutrition	53.02	2	<0.001	15.66 ^b^	8.51 ^a^	8.99 ^a^
NoGMO	101.22	2	<0.001	9.61 ^a^	21.31 ^b^	15.08 ^c^

**Table 4:** Key attribute model parameters (main effects): Price, Aspect, Certification, Origin, and Expectation. The statistics of the models can be consulted in Table A6 in Appendix B.

		Models’ Parameters (Significant Only)				
Models		Price	Aspect	Certification	Origin	Expectation
Age group						
	18–34	3.416		13.186		4.117
	35–54	3.538		11.585		4.734
	55–69	4.507		12.716		3.103
	≥70	7.006		17.758		2.998
Gender						
	Man		1.915			
	Woman		1.273			
Education						
	Primary	4.041				
	Basic	0.806				
	High School	0				
	BSc	0				
	MSc/PhD	0				
Children < 18						
	Yes			0		0
	No			−1.964		1.838
Buying place						
	Markets					1.928
	Dedicated stores					0
	Supermarkets					0
	Home sale					2.442
	Traditional stores					0

**Table 5:** (continuation 1 of Table 4) Key attribute model parameters (main effects): Buying easy, Health, Environment, Nutrition, and NoGMO. The statistics of the models can be consulted in Table A7 in Appendix B.

		Models’ Parameters (Significant Only)				
Models		Buying Easy	Health	Environment	Nutrition	NoGMO
Age group						
	18–34					
	35–54					
	55–69					
	≥70					
Gender						
	Man	2.583	25.539			20.382
	Woman	2.330	26.429			21.838
Education						
	Primary					
	Basic					
	High School					
	BSc					
	MSc/PhD					
Children < 18						
	Yes					
	No					
Buying place						
	Markets					−2.678
	Dedicated stores					0
	Supermarkets					0
	Home sale					0
	Traditional stores					0

**Table 6:** (continuation 2 of Table 4) Key attribute model parameters (2-way effects Country × Demographic variables): Price, Aspect, Certification, Origin, and Expectation. The statistics of the models can be consulted in Table A8 in Appendix B.

		Models’ Parameters (Interactions, Significant Only)				
Models		Price	Aspect	Certification	Origin	Expectation
Country × Age group						
	Greece × 18–34	3.462		−3.061		
	Greece × 35–54	0		−2.416		
	Greece × 55–69	0		−3.367		
	Greece × ≥70	0		−8.673		
	Spain × 18–34	0		0		
	Spain × 35–54	0		−3.266		
	Spain × 55–69	−2.438		0		
	Spain × ≥70	0		0		
	Portugal × 18–34	0		0		
	Portugal × 35–54	0		0		
	Portugal × 55–69	0		0		
	Portugal × 55–69	0		0		
Country × Gender						
	Greece × Men					4.564
	Greece × Women					3.012
	Spain × Men					0
	Spain × Women					−1.620
	Portugal × Men					0
	Portugal × Women					0
Country × Education						
	Greece × Primary		1.830		8.660	
	Greece × Basic		3.331		6.340	
	Greece × H. School		0		8.633	
	Greece × BSc		0		6.100	
	Greece × MSc		0		4.437	
	Spain × Primary		0		10.144	
	Spain × Basic		0		7.200	
	Spain × H. School		0		5.254	
	Spain × BSc		0		8.508	
	Portugal × Basic		0		3.547	
	Portugal × H. School		0		5.109	
	Portugal × BSc		0		8.508	
	Portugal × MSc/PhD		0		6.836	

**Table 7:** (continuation 3 of Table 4) Key attribute model parameters (2-way effects Country × Demographic variables): Buying easy, Health, Environment, Nutrition, and NoGMO. The statistics of the models can be consulted in Table A9 in Appendix B.

		Models’ Parameters (Interactions, Significant Only)				
Models		Buying Easy	Health	Environment	Nutrition	NoGMO
Country × Age group						
	Greece × 18–34					
	Greece × 35–54					
	Greece × 55–69					
	Greece × ≥70					
	Spain × 18–34					
	Spain × 35–54					
	Spain × 55–69					
	Spain × ≥70					
	Portugal × 18–34					
	Portugal × 35–54					
	Portugal × 55–69					
	Portugal × 55–69					
Country × Gender						
	Greece × Men	4.673		23.344	14.932	
	Greece × Women	3.151		25.705	16.377	
	Spain × Men	3.618		23.401	10.070	
	Spain × Women	3.160		23.769	9.871	
	Portugal × Men	0		25.010	10.447	
	Portugal × Women	0		25.271	9.499	
Country × Education						
	Greece × Primary		−6.705	−6.284		−8.322
	Greece × Basic		−3.202	0		−7.743
	Greece × H. School		−5.500	−5.035		−7.329
	Greece × BSc		0	0		−5.024
	Greece × MSc		0	0		−9.782
	Spain × Primary		0	1.476		0
	Spain × Basic		0	0		0
	Spain × H. School		0	2.379		0
	Spain × BSc		−1.523	0		−5.153
	Portugal × Basic		0	0		3.103
	Portugal × H. School		0	0		0
	Portugal × BSc		0	0		3.015
	Portugal × MSc/PhD		0	0		0

## Data Availability

The data used in this study are available upon reasonable request from the corresponding author only due to confidentiality restrictions.

## Abbreviations

The following abbreviations are used in this manuscript:

ANOVA	Analysis of variance
df	Degrees of freedom
LSD	Least significant differences
MANOVA	Multivariate analysis of variance
PCA	Principal components analysis

## Appendix A

**Table A1:** Table of contingency Country × Age.

Country × Age Crosstabulation											
		Age								Total	
		15–34		35–54		55–69		70+			
		N	%	N	%	N	%	N	%	N	%
Country	Greece	84	57.1%	75	20.2%	57	31.8%	34	65.4%	250	33.3%
	Spain	14	9.5%	154	41.4%	71	39.7%	11	21.2%	250	33.3%
	Portugal	49	33.3%	143	38.4%	51	28.5%	7	13.5%	250	33.3%
Total		147	100.0%	372	100.0%	179	100.0%	52	100.0%	750	100.0%

**Table A2:** Table of contingency Country × Gender.

Country × Gender Crosstabulation							
		Gender				Total	
		Male		Female			
		N	%	N	%	N	%
Country	Greece	115	40.5%	135	29.0%	250	33.3%
	Spain	83	29.2%	167	35.8%	250	33.3%
	Portugal	86	30.3%	164	35.2%	250	33.3%
Total		284	100.0%	466	100.0%	750	100.0%

**Table A3:** Table of contingency Country × Education level.

Country × Education Level Crosstabulation													
		Education Level										Total	
		Primary		Basic		High School		BSc		MSc/PhD			
		N	%	N	%	N	%	N	%	N	%	N	%
Country	Greece	120	96.8%	41	61.2%	54	61.4%	25	9.2%	10	5.1%	250	33.3%
	Spain	4	3.2%	21	31.3%	20	22.7%	205	75.1%	0	0.0%	250	33.3%
	Portugal	0	0.0%	5	7.5%	14	15.9%	43	15.8%	188	94.9%	250	33.3%
Total		124	100.0%	67	100.0%	88	100.0%	273	100.0%	198	100.0%	750	100.0%

**Table A4:** Table of contingency Country × Children < 18.

Country × Childreen < 18 Crosstabulation							
		Children < 18				Total	
		No		Yes			
		N	%	N	%	N	%
Country	Greece	141	35.1%	109	31.3%	250	33.3%
	Spain	128	31.8%	122	35.1%	250	33.3%
	Portugal	133	33.1%	117	33.6%	250	33.3%
Total		402	100.0%	348	100.0%	750	100.0%

**Table A5:** Table of contingency Country × Buying place.

Country × Buying Place Crosstabulation													
		Buying Place										Total	
		Markets		Dedicated Stores		Supermarkets		Home Sale		Traditional Stores			
		N	%	N	%	N	%	N	%	N	%	N	%
Country	Greece	44	21.5%	59	34.1%	89	52.7%	26	32.9%	32	25.8%	250	33.3%
	Spain	56	27.3%	53	30.6%	28	16.6%	37	46.8%	76	61.3%	250	33.3%
	Portugal	105	51.2%	61	35.3%	52	30.8%	16	20.3%	16	12.9%	250	33.3%
Total		205	100.0%	173	100.0%	169	100.0%	79	100.0%	124	100.0%	750	100.0%

## Appendix B

**Table A6:** Statistics of the key attribute models (main effects): Price, Aspect, Certification, Origin, and Expectation. The parameters of the models can be consulted in Table 4 of the main text.

		Models’ Statistics (χ^2^, df, *p*-Value)				
	Model	Price	Aspect	Certification	Origin	Expectation
Parameter						
All Model		530, 16, <0.001	486, 16, <0.001	731, 13, <0.001	683, 13, <0.001	670, 14, <0.001
Age group		8.11, 3, =0.044		14, 3, =0.003		9.0, 3, =0.03
	18–34	16, 1, <0.001		120, 1, <0.001		17, 1, <0.001
	35–54	43, 1, <0.001		302, 1, <0.001		34, 1, <0.001
	55–69	23, 1, <0.001		123, 1, <0.001		11, 1, =0.001
	≥70	9.2, 1, <0.001		37, 1, <0.001		5.2, 1, =0.022
Gender			7.4, 1, =0.007			
	Man		47, 1, <0.001			
	Woman		29, 1, <0.001			
Education		20.8, 4, <0.001				
	Primary	13, 1, <0.001				
	Basic	0.51, 1, 0.47				
	High School	1.6, 1, 0.20				
	BSc	0.05, 1, 0.82				
	MSc/PhD	Reference				
Children < 18						14, 1, <0.001
	Yes			Reference		Reference
	No			10, 1, <0.001		14, 1, <0.001
Buying place						13, 4, =0.011
	Markets					6.9, 1, =0.008
	Dedicated stores					<1, 1, > 0.05
	Supermarkets					1.5, 1, > 0.05
	Home sale					7.6, 1, =0.006
	Traditional stores					Reference

**Table A7:** Statistics of the key attribute models (main effects): Buying easy, Health, Environment, Nutrition, and NoGMO. The parameters of the models can be consulted in Table 5 of the main text.

		Models’ Statistics (χ^2^, df, *p*-Value)				
	Model	Buying Easy	Health	Environment	Nutrition	NoGMO
Parameter						
All Model		463, 6, <0.001	2054, 14, <0.001	2058, 16, <0.001	1003, 6, <0.001	1222, 18, <0.001
Age group						
	18–34					
	35–54					
	55–69					
	≥70					
Gender		550, 2, <0.001	5.1, 1, =0.024			7.6, 1, =0.006
	Man	18, 1, <0.001	1992, 1, <0.001			362, 1, <0.001
	Woman	29, 1, <0.001	2987, 1, <0.001			474, 1, <0.001
Education						
	Primary					
	Basic					
	High School					
	BSc					
	MSc/PhD					
Children < 18						
	Yes					
	No					
Buying place						17.3, 4, =0.002
	Markets					9.3, 1, =0.002
	Dedicated stores					<1, 1, > 0.05
	Supermarkets					1.7, 1, > 0.05
	Home sale					3.6, 1, > 0.05
	Traditional stores					Reference

**Table A8:** Statistics of the key attribute models (2-way effects Country × Demographic variables): Price, Aspect, Certification, Origin, and Expectation. The parameters of the models can be consulted in Table 6 of the main text.

		Models’ Statistics (χ^2^, df, *p*-Value) Interactions				
	Model	Price	Aspect	Certification	Origin	Expectation
Parameter						
Country × Age group		23, 8, =0.003		39, 8, <0.001		
	Greece × 18–34	6.5, 1, =0.011		5.0, 1, =0.025		
	Greece × 35–54	1.7, 1, >0.05		5.0, 1, =0.026		
	Greece × 55–69	3.3, 1, >0.05		5.3, 1, =0.022		
	Greece × ≥ 70	<1, 1, >0.05		7.6, 1, =0.006		
	Spain × 18–34	1.2, 1, >0.05		2.1, 1, >0.05		
	Spain × 35–54	<1, 1, >0.05		14, 1, <0.001		
	Spain × 55–69	4.1, 1, =0.042		<1, 1, >0.05		
	Spain × ≥ 70	<1, 1, >0.05		<1, 1, >0.05		
	Portugal × 18–34	Reference		Reference		
	Portugal × 35–54	Reference		Reference		
	Portugal × 55–69	Reference		Reference		
	Portugal × 55–69	Reference		Reference		
Country × Gender						84, 5, <0.001
	Greece × Men					34, 1, <0.001
	Greece × Women					17, 1, <0.001
	Spain × Men					1.1, 1, >0.05
	Spain × Women					5.4, 1, =0.02
	Portugal × Men					1.0, 1, >0.05
	Portugal × Women					Reference
Country × Education			219,12, <0.001		1114,13, <0.001	
	Greece × Primary		111,1, <0.001		191, 1, <0.001	
	Greece × Basic		12, 1, <0.001		35, 1, <0.001	
	Greece × H. School		48, 1, <0.001		85, 1, <0.001	
	Greece × BSc		3, 1, >0.05		20, 1, <0.001	
	Greece × MSc		<1, 1, >0.05		4, 1, =0.041	
	Spain × Primary		<1, 1, >0.05		9, 1, =0.003	
	Spain × Basic		1.2, 1, >0.05		23, 1, <0.001	
	Spain × H. School		1.1, 1, >0.05		12, 1, <0.001	
	Spain × BSc		3.8, 1, >0.05		473, 1, <0.001	
	Portugal × Basic		Reference		1.3, 1, >0.05	
	Portugal × H. School		Reference		7.8, 1, <0.001	
	Portugal × BSc		Reference		66, 1, <0.001	
	Portugal × MSc/PhD		Reference		187, 1, <0.001	

**Table A9:** Statistics of the key attribute models (2-way effects Country × Demographic variables): Buying easy, Health, Environment, Nutrition, and NoGMO. The parameters of the models can be consulted in Table 7 of the main text.

		Models’ Statistics (χ^2^, df, *p*-Value) Interactions				
	Model	Buying Easy	Health	Environment	Nutrition	NoGMO
Parameter						
Country × Age group						
	Greece × 18–34					
	Greece × 35–54					
	Greece × 55–69					
	Greece × ≥70					
	Spain × 18–34					
	Spain × 35–54					
	Spain × 55–69					
	Spain × ≥70					
	Portugal × 18–34					
	Portugal × 35–54					
	Portugal × 55–69					
	Portugal × 55–69					
Country × Gender		71, 4, <0.001		9.7, 3, =0.022	2107, 6, <0.001	
	Greece × Men	35, 1, <0.001		139, 1, <0.001	488, 1, <0.001	
	Greece × Women	24, 1, <0.001		175, 1, <0.001	689, 1, <0.001	
	Spain × Men	18, 1, <0.001		1134, 1, <0.001	160, 1, <0.001	
	Spain × Women	27, 1, <0.001		2248, 1, <0.001	310, 1, <0.001	
	Portugal × Men	Reference		1213, 1, <0.001	179, 1, <0.001	
	Portugal × Women	Reference		2458, 1, <0.001	282, 1, <0.001	
Country × Education			129, 12, <0.001	33, 10, <0.001		175, 12, <0.001
	Greece × Primary		88, 1, <0.001	9.9, 1, =0.002		90, 1, <0.001
	Greece × Basic		9.3, 1, =0.002	1.8, 1, >0.05		37, 1, <0.001
	Greece × H. School		34, 1, <0.001	5.8, 1, =0.016		40, 1, <0.001
	Greece × BSc		1.9, 1, >0.05	<1, 1, >0.05		10, 1, =0.001
	Greece × MSc		<1, 1, >0.05	Reference		16, 1, <0.001
	Spain × Primary		<1, 1, >0.05	<1, 1, >0.05		<1, 1, >0.05
	Spain × Basic		<1, 1, >0.05	3.9, 1, =0.049		2.0, 1, >0.05
	Spain × H. School		<1, 1, >0.05	2.8, 1, >0.05		<1, 1, >0.05
	Spain × BSc		6.1, 1, =0.013	Reference		44, 1, <0.001
	Portugal × Basic		1.0, 1, >0.05	1.1, 1, >0.05		<1, 1, >0.05
	Portugal × H. School		<1, 1, >0.05	<1, 1, >0.05		<1, 1, >0.05
	Portugal × BSc		<1, 1, >0.05	<1, 1, >0.05		5.9, 1, =0.015
	Portugal × MSc/PhD		Reference	Reference		Reference
